# Disentangling conscious and unconscious processing: a subjective trial-based assessment approach

**DOI:** 10.3389/fnhum.2013.00769

**Published:** 2013-11-18

**Authors:** Eva Van den Bussche, Astrid Vermeiren, Kobe Desender, Wim Gevers, Gethin Hughes, Tom Verguts, Bert Reynvoet

**Affiliations:** ^1^Faculty of Psychology and Educational Sciences, Department of Psychology, Vrije Universiteit BrusselBrussels, Belgium; ^2^Unité de recherche Conscience, Cognition et Computation, Université Libre de BruxellesBrussels, Belgium; ^3^Unité de Recherché en Neurosciences Cognitives, Université Libre de BruxellesBrussels, Belgium; ^4^Department of Psychology, University of EssexEssex, UK; ^5^Department of Experimental Psychology, Ghent UniversityGhent, Belgium; ^6^Department of Psychology, University of LeuvenLeuven, Belgium

**Keywords:** conscious processing, unconscious processing, prime awareness, stimulus strength, awareness assessment

## Abstract

The most common method for assessing similarities and differences between conscious and unconscious processing is to compare the effects of unconscious (perceptually weak) stimuli, with conscious (perceptually strong) stimuli. Awareness of these stimuli is then assessed by objective performance on prime identification tasks. While this approach has proven extremely fruitful in furthering our understanding of unconscious cognition, it also suffers from some critical problems. We present an alternative methodology for comparing conscious and unconscious cognition. We used a priming version of a Stroop paradigm and after each trial, participants gave a subjective rating of the degree to which they were aware of the prime. Based on this trial-by-trial awareness assessment, conscious, uncertain, and unconscious trials were separated. Crucially, in all these conditions, the primes have identical perceptual strength. Significant priming was observed for all conditions, but the effects for conscious trials were significantly stronger, and no difference was observed between uncertain and unconscious trials. Thus, awareness of the prime has a large impact on congruency effects, even when signal strength is controlled for.

## INTRODUCTION

It is now generally accepted that primes that are not consciously perceived can nevertheless be processed by the cognitive system at high, semantic processing levels (for a review see Van den Bussche et al., 2009a,b; Van Opstal et al., 2010). Accumulating evidence has demonstrated the far-reaching capabilities of unconscious processing, even targeting high-level cognitive functions such as response inhibition (e.g., van Gaal et al., 2008; Hughes et al., 2009), task-switching (e.g., Lau and Passingham, 2006), context setting (Van Opstal et al., 2011), and post-error slowing (i.e., remedial action to prevent future errors; Cohen et al., 2009). These various demonstrations that unconscious processing can reach high processing levels brings to attention the important question whether unconscious processing has limits, and if so then what is the specific function of consciousness (Dehaene, 2008; Lau, 2009).

A standard procedure to test the contribution of consciousness in a cognitive process is to test whether the cognitive process can also be carried out when subjects remain unaware of the critical stimuli. The reasoning behind this approach is straightforward. If an effect is selectively present with conscious stimuli, this shows that consciousness is a prerequisite for the cognitive process underlying the effect. Based on this simple logic, several studies have claimed that the cognitive processes involved in the detection of errors (Nieuwenhuis et al., 2001), in the resolution of conflict (Kunde, 2003; Ansorge et al., 2011), or in the adaptation to a critical format manipulation (Van den Bussche et al., 2008) or a manipulation of the congruency proportions (Merikle and Joordens, 1997) are selectively associated with conscious processing.

Typically, researchers examine whether a certain cognitive process can be carried out on clearly visible stimuli, and, to test the involvement of consciousness, additionally test whether the cognitive process can also be carried out when the stimulus remains unconscious. In order to create unconscious conditions, the critical stimuli are very briefly presented and heavily masked, which prohibits conscious processing of the stimulus. Therefore, it is impossible for subjects to notice the presence of such stimuli. When this technique is used, subjects are typically unaware of the stimuli. If the mask is removed, visibility of the stimuli increases drastically. Importantly, in addition to a sharp reduction in awareness of the critical stimulus, this procedure also sharply reduces the signal strength of the critical stimulus (Lau, 2009). Consequently, smaller effects with masked compared to unmasked stimuli (e.g., Dehaene et al., 2003; van Gaal et al., 2010) might just be the result of a difference in signal strength, rather than a difference in awareness.

In order to confirm that the manipulation of signal strength was successful to reduce prime visibility, many experimenters collect an objective measure of prime visibility. Following the main experiment, in which the instruction is typically to classify the targets, subjects are now asked to categorize the primes. Based on signal detection theory, a *d*′ measure (Green and Swets, 1966) is calculated based on these data, which is taken as an indication of subjects’ sensitivity for the primes. Typically one level of the primes is treated as signal and the other as noise. A *d*′ measure is then computed as the difference between the proportion of hits and the proportion of false alarms. In this *post-hoc* test, subjects typically perform worse in categorizing the stimuli when the signal strength is severely reduced, compared to when it is not. When subjects are at chance level in categorizing heavily masked stimuli (i.e., *d*′ = 0) this is often taken as a reflection that subjects were unaware of the stimuli. Although this approach has proven extremely valuable in furthering our understanding of unconscious cognition, it also has some drawbacks. For example, *d*′ might be overestimated because of unconscious influences on the post-experiment awareness test (i.e., as shown in blind sight patients; Stoerig and Cowey, 1997, 2007; Weiskrantz, 2009) or because of perceptual learning throughout the main experiment which is expressed in elevated performance during the posttest (Schlaghecken et al., 2008). Alternatively, *d*′ might be underestimated due to testing condition artifacts. Because the posttest is always administered following the main experiment, fatigue might play an important role, and because detecting heavily masked stimuli is much more difficult than detecting clearly visible stimuli, subjects may lose motivation throughout the posttest (Pratte and Rouder, 2009; but see Finkbeiner, 2011 for a rebuttal). Furthermore, a sizable number of trials is needed to assure sufficient statistical power to reject the hypothesis of *d*′ being larger than zero (Amihai, 2012), while in practice, researchers tend to use fewer trials for the awareness test than for the priming blocks.

There is a close connection between the problem of signal strength and the problem of using a *d*′ obtained in a post-experiment detection task as an index of awareness. The reduction of *d*′ with masked stimuli in the posttest might just express an overall reduction in signal strength rather than a reduction in awareness. Consequently, the absence of an effect in the main experiment with *primes which are categorized at chance level in the posttest* might just reflect the necessity of sufficient signal strength to produce the effect. In a similar vein, the presence of an effect with *unconscious* primes might just reflect an insensitivity of our posttest *d*′ measure towards partial awareness of masked stimuli (Kouider and Dupoux, 2004).

### A TRIAL-BASED ASSESSMENT APPROACH

In the current study, we tried to solve this complex issue by abandoning both problematic constructs. Because we cannot equate the *d*′ obtained in the posttest with awareness, we need another approach to reliably classify primes as either consciously or not consciously perceived. For example, Lau and Passingham (2006) showed that subjective reports of stimulus awareness can vary heavily in two conditions with a perfectly matched *d*′. The authors emphasized that their findings showed that *d*′ is just a measure of signal-to-noise ratio, and that the most straightforward way to examine whether subjects are aware of a stimulus or not is to simply ask them (Dehaene, 2008; Lau, 2009). After all, the main determinant of awareness is the introspective judgment that we are aware of a stimulus (Cleeremans, 2011). If we are not aware that a stimulus was presented at all, it seems appropriate to label this stimulus as unconscious.

Although subjective measures have not always been treated as valuable data in cognitive sciences (Jack and Roepstorff, 2002), or even have a problematic relation with it (Nisbett and Wilson, 1977), convincing evidence exists that subjects can accurately introspect their own awareness of visual information (Corallo et al., 2008; Marti et al., 2010). As convincingly argued by Hurlburt and Heavey (2001), problems associated with introspective methods of awareness, such as the under confidence phenomenon (Björkman et al., 1993), should not be used to disapprove introspection *per se*, but rather as a means to develop better introspective measures. When using appropriate measures, such as the perceptual awareness scale (i.e., PAS; Ramsøy and Overgaard, 2004), it has been shown that subjects can exhaustively report on their conscious experiences (Sandberg et al., 2010). Following the suggestion to use subjective prime awareness reports on a single-trial basis (Cheesman and Merikle, 1984; Dehaene, 2008; Lau, 2009; Dehaene and Changeux, 2011), we present a paradigm in which awareness of the primes is not *a priori* specified based on manipulating the signal strength, but rather is derived from the subjective judgment of awareness. The aim of the current paper is not to suggest abolishing objective prime awareness measures or to provide a perfect measure of prime awareness, but rather to explore alternative measures where consciousness is inferred from introspection and where visual strength in conscious and unconscious conditions is perfectly matched. This could stimulate new insights and debate.

In our paradigm, we will present primes for the same intermediate duration (i.e., 40 ms) throughout the entire experiment. Because extensive pilot testing showed that the threshold of consciousness varies around this value, subjects will be aware of the masked primes on some trials and unaware on others. Because stimulus conditions are completely identical in both situations, our paradigm allows creating conscious and unconscious conditions while keeping stimulus strength constant. As such, the paradigm can be used to reexamine a whole range of questions (e.g., comparing conscious and unconscious priming; comparing conscious and unconscious conflict adaptation). In our study, we used a priming version of the Stroop paradigm, in which primes are color words printed in black, and targets are colored symbols. Additionally, there are several reasons why we chose this task over other alternatives. First, because in our modified Stroop task primes (i.e., words) and targets (i.e., colors) trigger the same response while being perceptually dissimilar, sensory identity priming can be ruled out as a possible explanation of our results. Second, and most important, a huge advantage of this perceptual dissimilarity between prime and target is that it is much easier for subjects to judge whether they were aware of the primes or not. If prime and target are perceptually identical, subjects often discard weak prime information as being caused by the target (Vermeiren and Cleeremans, 2012). If prime information is perceptually completely separated from target information, as is the case in our study, it is much clearer for subjects whether or not they were aware of the primes.

Although a trial-by-trial prime awareness assessment has been used before in combination with a target task, previous studies either did not use it to contrast conscious and unconscious trials (e.g., Klotz and Neumann, 1999) or did not match stimulus strength (e.g., Ansorge et al., 2011). Using the trial-based assessment approach described above, we will be able to differentiate three types of trials with matched stimulus strength: *conscious* trials, where participants correctly identified the prime and were certain of the prime identity; *uncertain* trials, where participants correctly identified the prime, but were uncertain of the prime identity; *unconscious* trials where the participants reported not being able to identify the prime. For these three conditions, we will examine whether priming effects were observed and how these effects differ between conditions.

## MATERIALS AND METHODS

### PARTICIPANTS

Fifty-six students (nine male) with a mean age of 19.0 years (SD = 1.50, range 17–25) participated as partial fulfilment of a course requirement.

### STIMULI

Stimuli were presented on a 75-Hz, 15′′ color screen connected to a computer running the Windows operating system in a dimly lit room. Stimulus delivery and the recording of reaction time and accuracy were controlled by E-prime (; Psychology Software Tools). All stimuli appeared in Arial 14 pt. The primes, between 2.3 and 2.7 cm wide and 0.7 cm high, were either the color word “ROOD” (“red” in Dutch) or the color word “BLAUW” (“blue” in Dutch), presented as black uppercase letters on a white background. The targets consisted of five symbols (&&&&&) printed either in red or blue. This led to four prime-target combinations (two primes x two targets). Half of the trials were congruent (i.e., prime and target elicited the same response) and half were incongruent (i.e., prime and target elicited different responses). Stimulus sequences were randomly generated.

### PROCEDURE

**FIGURE 1:**
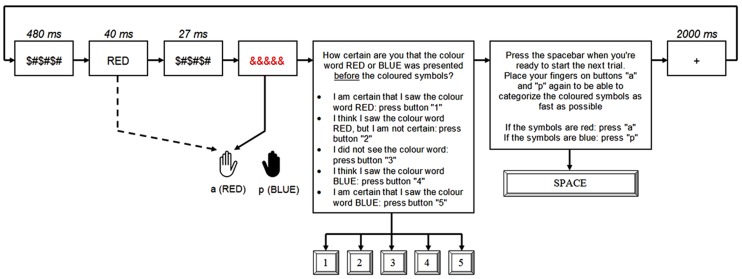
**Example of a congruent trial**.

**Figure 1** depicts the sequence of a trial. All stimuli appeared in the center of the screen. First, a forward mask ($#$#$#) was shown for 480 ms, followed by a prime presented for 40 ms. The prime was followed by a backward mask ($#$#$#) for 27 ms. Finally, a target was presented until the participants’ response was registered. Before the experiment started, subjects were carefully debriefed about the structure of the experiment, with the emphasis on the fact that a color word (either “RED” or “BLUE”), printed in black, will be presented before the colored symbols. They were told to respond as fast as possible to the color of the target symbols, and afterward to indicate whether they had seen the identity of the prime on that trial or not.

Following their response to the target, subjects were asked to indicate how certain they were about the identity of the colur word prime on the current trial using a five-point PAS (Ramsøy and Overgaard, 2004) ranging from 1 to 5: “I am certain that I saw the color word RED: press “1” on the keyboard;” “I think I saw the color word RED, but I am not certain: press “2” on the keyboard;” “I did not see the color word: press “3” on the keyboard;” “I think I saw the color word BLUE, but I am not certain: press “4” on the keyboard;” “I am certain that I saw the color word BLUE: press ‘5’ on the keyboard.” Subjects could take as much time as needed for this second task.

After this subjective judgment of prime awareness, subjects were shown the instructions again, and asked to press the spacebar when ready, which initiated the next trial. Once they pressed the spacebar, a plus sign (+) was shown for 2000 ms, after which a new trial started. The experiment started with 32 practice trials on which they received feedback following each trial about their performance on the speeded response task. Afterward, the main experiment started. Each subject received four blocks, consisting of 80 trials. All presentations were synchronized with the vertical refresh cycle of the screen (13.3 ms).

## RESULTS

### SELECTION OF PARTICIPANTS

In a first step, only those participants that were eligible to be included in the analyses were selected. Three exclusion criteria were used. First, in order to conduct reliable analyses, a sufficient number of trials had to be available in each of the conditions under investigation (conscious, uncertain, and unconscious condition) for each participant. To achieve this, all participants with less than 15 trials in one or more of these conditions were excluded (*N* = 16). Second, participants with a guessing strategy were excluded. When participants were able to identify the primes, the proportion of correct prime identifications should be clearly above chance level (i.e., 50%). If the proportion of correct prime identifications is close to chance level, this indicates that the participant was guessing instead of being able to identify the primes. Therefore, participants whose proportion of correct prime identifications (based on scores 1, 2, 4, and 5) was below 55% were excluded (*N* = 2). Third, participants who made more than 20% errors in their response to the targets were omitted (*N* = 1). In total, this led to the exclusion of 19 of the 56 participants. The final sample consisted of 37 participants (seven males) with a mean age of 19.0 years (SD = 1.42, range 18–24).

### CONSCIOUS, UNCERTAIN, AND UNCONSCIOUS TRIALS

Looking at the proportion of correctly identified primes, it became apparent that there was a clear difference between trials where participants indicated being certain of the prime identity by giving a score of “1” or “5” on the PAS scale and trials where participants indicated being uncertain of the prime identity by giving a score of “2” or “4” on the PAS scale. More specifically, the proportion of correctly identified primes was significantly higher when they were certain of the prime identity compared to when they were uncertain [88 versus 73%, *t*(36) = 11.32, *p* < 0.001].

For the analyses, trials where participants correctly identified the prime and were certain of the prime identity (score “1” or “5” on the PAS scale) were labeled as “conscious” trials. Trials where participants correctly identified the prime, but were uncertain of the prime identity (score “2” or “4” on the PAS scale) were labeled as “uncertain” trials. “Unconscious trials” were labeled as those trials where the participants reported not being able to identify the prime (score “3” on the PAS scale). Trials where participants reported having seen a blue prime when the prime was in fact red (on average 8.6%) or a red prime when the prime was in fact blue (on average 5.9%) were excluded from analyses. Because these latter errors occurred so infrequently, no reliable analyses could be conducted on these trials. After additionally excluding trials where an error was made on the response to the target (see below), this led to an average of 103 conscious trials (range 17–267), 87 uncertain trials (range 18–162), and 61 unconscious trials (range 16–199) across subjects. **Table 1** provides an overview of the number of trials in each condition per subject.

**Table 1:** Number of included trials in each condition (conscious, uncertain, or unconscious) per subject and the overall mean.

Subject	Conscious	Uncertain	Unconscious
1	69	73	58
2	151	95	38
3	169	34	19
4	92	113	89
5	121	19	106
6	102	104	56
7	41	81	105
8	267	19	19
9	174	38	16
10	34	101	58
11	90	104	65
12	147	104	22
13	129	127	38
14	18	137	105
15	17	70	199
16	122	74	37
17	69	102	21
18	119	84	31
19	218	45	26
20	32	136	104
21	36	98	100
22	63	104	122
23	137	18	48
24	90	85	62
25	18	112	162
26	99	162	17
27	85	55	91
28	76	129	62
29	202	34	34
30	65	140	50
31	127	126	26
32	150	79	39
33	88	114	66
34	207	30	39
35	78	63	33
36	25	111	58
37	79	116	39
Mean	103	87	61

### RT ANALYSES

Erroneous responses to the target (on average 7.3%) were excluded. Median RTs from correct responses to the target were submitted to a repeated measures analysis with condition (conscious, uncertain, or unconscious) and congruency (congruent or incongruent) as within-subject factors. Mean of median RTs as a function of these factors are reported in **Table 2**. The main effect of congruency was significant [*F*(1,36) = 41.45, *p* < 0.001]. On average, participants responded 74 ms slower to incongruent than congruent trials. The main effect of condition was also significant [*F*(2,35) = 15.23, *p* < 0.001]: average RTs were 606 ms on conscious trials, 549 ms on uncertain trials, and 575.5 ms on unconscious trials. The factors condition and congruency significantly interacted [*F*(2,35) = 12.54, *p* < 0.001]: the observed congruency effect (RT on incongruent trials – RT on congruent trials) was significantly different for conscious, uncertain, and unconscious trials (respectively, 140, 48, and 33 ms). One sample *t*-tests indicated that all congruency effects significantly differed from zero [*t*(36) = 7.31, *p* < 0.001 for conscious trials, *t*(36) = 2.58, *p* = 0.014 for uncertain trials and *t*(36) = 2.05, *p* = 0.048 for unconscious trials]. Paired samples *t*-tests indicated that congruency effects were significantly larger for conscious trials than for uncertain [*t*(36) = 4.54, *p* < 0.001] and unconscious trials [*t*(36) = 4.02, *p* = 0.001]. No difference was observed between the congruency effects for uncertain and unconscious trials [*t*(36) = -0.59, *p* = 0.56].

**Table 2:** Mean (SD) of the median RTs (in ms) and mean error rates (in %) as a function of condition and congruency and the congruency effects (incongruent-congruent).

	Congruent	Incongruent	Congruency effect
**Conscious**			
RT	536 (153.1)	676 (214.9)	140^***^
Error %	2.7 (3.1)	15.3 (14.1)	12.6^***^
**Uncertain**			
RT	525 (151.9)	573 (221.2)	48^*^
Error %	3.6 (5.8)	5.6 (4.8)	2.0°
**Unconscious**			
RT	559 (195.2)	592 (220.4)	33^*^
Error %	10.1 (9.5)	14.0 (11.9)	3.9^*^

*** p < 0.001

* p < 0.05

° p < 0.08.

### ERROR ANALYSES

The same repeated measures analysis was performed on mean error rates. Mean error rates as a function of condition and congruency are reported in **Table 2**. This analysis revealed a significant main effect of congruency [*F*(1,36) = 31.54, *p* < 0.001]. On average, participants made 6.1% more errors on incongruent than congruent trials. The main effect of condition was also significant [*F*(2,35) = 21.07, *p* < 0.001]: average error rates were 9.4% on conscious trials, 4.6% on uncertain trials and 12.0% on unconscious trials. The factors condition and congruency significantly interacted [*F*(2,35) = 11.41, *p* < 0.001]: the observed congruency effect (errors on incongruent trials – errors on congruent trials) was significantly different for conscious, uncertain and unconscious trials (respectively, 12.6, 2.0, and 3.9%). One sample *t*-tests indicated that the congruency effects for conscious and unconscious trials significantly differed from zero [respectively, *t*(36) = 5.30, *p* < 0.001 and *t*(36) = 2.12, *p* = 0.041]. The congruency effect for uncertain trials was only marginally significant [*t*(36) = 1.90, *p* = 0.065]. Paired samples *t*-tests indicated that congruency effects were significantly larger for conscious trials than for uncertain [*t*(36) = 4.78, *p* < 0.001] and unconscious trials [*t*(36) = 2.81, *p* = 0.008]. No difference was observed between the congruency effects for uncertain and unconscious trials [*t*(36) = 0.86, *p* = 0.40].

## DISCUSSION

In the current study, we used a paradigm to reliably disentangle conscious and unconscious processing based on a trial-by-trial prime awareness assessment. In traditional masking paradigms, unconscious primes typically have a lower signal strength than conscious primes, confounding the comparison between conscious and unconscious processing. As an alternative approach, we categorized trials with identical stimulus strength as either conscious, uncertain, or unconscious based on the trial-by-trial prime awareness assessment. With this paradigm, we observed significant priming for conscious, uncertain, and unconscious trials. However, the effects for conscious trials were significantly stronger than the effects of the other two types. These quantitative differences show that this paradigm is able to distinguish conscious from uncertain and unconscious processing. No significant difference was observed between the uncertain trials, where participants made correct but uncertain prime identifications, and unconscious trials, where participants claimed they did not see the primes. These results show that even when the signal strength is kept constant, primes which are consciously perceived cause much larger effects than primes which cause uncertainty or even remain subjectively unconscious (Haynes et al., 2005).

### MATCHING STIMULUS STRENGTH

The problematic difference in signal strength between conscious and unconscious trials has already received some attention in the literature (Lau, 2009). A few studies already tried to match for differences in signal strength, and examined the influence of this matching on measures of awareness. For example, Lamy et al. (2009) also presented stimuli for an intermediate duration followed by a mask, and categorized trials as either conscious or unconscious based on a trial-by-trial awareness measure. Using EEG, these authors confirmed the observation of previous studies that the amplitude of the P3 was larger in the conscious compared to the unconscious condition. Among similar lines, a few studies matched for differences in signal strength between conscious and unconscious conditions, when studying conscious access (Ramsøy and Overgaard, 2004; Sergent and Dehaene, 2004; Del Cul et al., 2007). Importantly, the crucial addition of the current paradigm with respect to previous work is that in our paradigm the trial-based assessment method is used to create conscious and unconscious trials with identical signal strength, while at the same time we are gathering information on the masked priming task. By doing this, we can combine the trial-based visibility information with traditional measures of cognitive processing (e.g., priming effects). Thus, with the current paradigm we can examine whether previous studies which observed that certain cognitive functions require conscious processing (Nieuwenhuis et al., 2001; Kunde, 2003) were actually the result of a difference in signal strength or not, which contributes to the study of the function of consciousness. Although two recent studies have also tried to accomplish this, they did not control the stimulus strength in the conscious and unconscious conditions (Ansorge et al., 2011) or used a dichotomous prime awareness measure (Ro et al., 2009) which is unable to exhaustively measure all conscious knowledge that is available for the subject (i.e., exhaustiveness), while excluding unconscious knowledge (i.e., exclusiveness; Sandberg et al., 2011).

### THE MAGNITUDE OF PRIMING EFFECTS IS DETERMINED BY PRIME AWARENESS

The results of the current study directly oppose those of Francken et al. (2011), who claimed that priming effects are independent of prime awareness. Using either a metacontrast mask (which effectively masked the primes) or a pseudo mask (which looked similar to the former, but was inefficient in masking the prime), large differences in discriminability of the primes were observed between these two conditions. Notwithstanding this difference in discriminability, priming effects were completely identical. According to the authors, the absence of a difference in priming effects between both types of masks could be taken as evidence that the signal strength was matched (Francken et al., 2011), and thus that priming effects are independent of prime awareness once signal strength is controlled for. However, this finding was probably caused by the fact that both masks did not render stimuli completely unconscious, and that the true difference in visibility between both masks was much smaller than the suggested difference (Desender and Van den Bussche, 2012). In our opinion, to fully match the signal strength of a conscious and an unconscious condition, presentation conditions should be identical in both cases, as is the case in the current study. Only then, differences in signal strength as an underlying cause can completely be ruled out as an alternative for differences in effects dependent on prime awareness.

### PROS AND CONS

Apart from the clear methodological improvements of the current paradigm, a major advantage is that it can be particularly well utilized in combination with brain imaging methods as it allows the concurrent assessment of factors that influence prime awareness and the processes involved in unconscious priming. For example, many recent studies have focused on identifying pre-stimulus EEG predictors of conscious recognition of a stimulus such as the amplitude and coherence of EEG oscillations (Hanslmayr et al., 2007) or the ongoing phase of alpha activity (Busch et al., 2009). By combining trial-to-trial objective and subjective awareness tests with a priming task, future studies may be able to determine if similar factors predict the magnitude of priming as well as assessing the interaction between EEG measures of both prime visibility and priming. Such an approach could significantly further our understanding of how unconscious processes are implemented in the brain, and thereby help validate or refute different theoretical approaches to understanding consciousness.

The current paradigm also has certain drawbacks. Because of the exclusion criteria, it requires a large number of participants to reach sufficient power. Introspective methods also give rise to variance in the number of trials in each condition. Since the participants themselves indicate which trials are conscious, uncertain, and unconscious, this will differ strongly between participants (see **Table 1**). In order to check whether this variance had a strong influence on the observed priming effects, we divided the participants in three groups based on whether their majority of trials was conscious, uncertain, or unconscious. There were no differences in the RT analysis between participants with mostly conscious trials, mostly uncertain trials and mostly unconscious trials for each of the observed priming effects (all *p* > 0.39). In the error rate analysis, there was only a difference between the three groups for the priming effect for conscious trials [*F*(2,34) = 6.54, *p* = 0.004]: the priming effect for conscious trials was larger for participants with mostly uncertain (20.0%) or unconscious (18.6%) trials compared to participants with mostly conscious trials (4.5%). This latter effect could be due to the fact that participants with mostly uncertain or unconscious trials are more conservative in deciding when a trial was truly conscious and only do this on trials where the prime was extremely visible for them, which could lead to stronger priming effects. These results indicate that, although introspective methods inherently lead to variability in the distribution of the data, this seemed to have only a limited influence on the observed pattern of results.

Additionally, participants probably used varying strategies to “categorize” their phenomenological experience of the prime using the discrete response scale. For example, some participants might use a higher threshold to judge a prime as “not seen” compared to other participants. This can also induce variability in the number of trials per condition across participants. In future studies, this source of variance should be avoided, for example by using a more continuous scale (and thus not forcing participants to judge their experience in a discrete manner; e.g., Todd et al., 2012), by training participants on the ability to introspect (e.g., Lutz and Thompson, 2003), or by combining a subjective with an objective prime awareness measure. For example, in a recent study Morsella et al. (2009) wanted to assess the subjective experience associated with sustaining incompatible intentions. In order to assure that all subjects introspected “the same thing,” they were first trained in a separate task to associate the experience associated with an incompatible event to a corresponding response.

Furthermore, one might argue that in this paradigm the subjects’ attention is drawn towards the presence of the primes. Participants are aware of the primes from the start, which probably increases their attention to the primes and thus perhaps their prime awareness. However, in this case the awareness level of the primes, although heightened, always remains stable, whereas in typical masked priming experiments participants are not informed about the primes during the priming experiment, but only during the *post-hoc* prime visibility assessment, which would lead to a difference in the awareness of the primes at the time the priming effects are measured and at the time the prime awareness is assessed. One could also argue that during uncertain and unconscious trials participants’ degree of attention is lower (e.g., due to mind-wandering) causing decreased priming effects. Indeed, we did not control for effects such as mind-wandering, but this is true for almost all traditional priming research as well. Still, future studies (using both traditional and trial-by-trial prime awareness assessment) should take this into account. Another potential drawback is the fact that the paradigm induces a dual task context: participants first respond to the target and afterwards perform a task on the prime. This creates a different task situation compared to traditional priming paradigms. Although this did not impair masked priming in the current study, it might influence the observed results under certain circumstances (e.g., Fischer et al., 2011). The current paradigm is also unable to disentangle the causality of priming and prime identification. One might argue that, intuitively, a higher prime visibility will induce stronger priming effects. However, it could also be the case that trials where a strong priming effect is present lead to some metacognitive awareness (e.g., awareness of speed of RTs, awareness of the urge to press the wrong response button, a notion of the difficulty of the trial; e.g., Marti et al., 2010) and this in turn might aid prime identification.

## CONCLUSION

We conclude that even when perfectly matching stimulus strength in conscious and unconscious conditions based on a trial-based assessment of prime awareness, much stronger priming effects are observed for conscious versus unconscious or uncertain trials. This method proves to be a fruitful approach to disentangle conscious and unconscious processing. Still, more work has to be done. Primarily, as suggested in the discussion, future studies should focus on minimizing the variability between participants which occurs when they introspect on their phenomenological experience. We should continue to search for ways to optimally integrate phenomenology in cognitive science.

## Conflict of Interest Statement

The authors declare that the research was conducted in the absence of any commercial or financial relationships that could be construed as a potential conflict of interest.
